# Developing a yeast cell factory for the production of terpenoids

**DOI:** 10.5936/csbj.201210006

**Published:** 2012-11-05

**Authors:** Sotirios C. Kampranis, Antonios M. Makris

**Affiliations:** a School of Medicine, University of Crete, P.O. Box 2208, Heraklion 71003, Greece; b Institute of Applied Biosciences/ CERTH, P.O. Box 60361, Thermi 57001, Thessaloniki, Greece

## Abstract

Technological developments over the past century have made microbes the work-horses of large scale industrial production processes. Current efforts focus on the metabolic engineering of microbial strains to produce high levels of desirable end-products. The arsenal of the contemporary metabolic engineer contains tools that allow either targeted rational interventions or global screens that combine classical approaches with –omics technologies. Production of terpenoids in S. cerevisiae presents a characteristic example of contemporary biotechnology that integrates all the variety of novel approaches used in metabolic engineering. Terpenoids have attracted significant interest as pharmaceuticals, flavour and fragrance additives, and, more recently, biofuels. The ongoing metabolic engineering efforts, combined with the continuously increasing number of terpene biosynthetic enzymes discovered will enable the economical and environmentally friendly production of a wide range of compounds.

## Introduction

Since antiquity, microbial fermentation processes have been extensively used for the processing of foods and the production of beverages, while technological developments over the past century made microbes the work-horses for large scale industrial production processes. Since the 1980s in particular, significant advances in genetic engineering have converted microbes to “cell factories” for the production of a diverse range of important chemical compounds. Manipulation of microbial metabolism holds major advantages, since microbes offer an environmentally friendly means to efficiently convert cheap raw materials like glucose, sucrose, and biomass derived materials into high value chemicals and fuels. *Saccharomyces cerevisiae* is an organism highly preferred by the industry, as it can withstand high osmotic pressure and reduced pH compared to bacteria [1]. Currently, there is continuous development and improvement of yeast strains for the production of high levels of desirable end products. The pace of strain development has accelerated as new tools for metabolic engineering manipulations are introduced. The overall approach for generating high production strains is currently based on a number of complementary approaches that include: a) the upregulation of desirable biosynthetic pathways, b) the suppression of pathways that drain resources or precursors (competing pathways), c) the introduction of exogenous genes or biosynthetic pathways, and d) the development of methodologies to alleviate stress and/or toxicity caused by the production of high levels of the product or of an undesirable intermediate. The arsenal of the contemporary metabolic engineer contains tools that allow for either targeted rational interventions that introduce changes in the strain's genotype based on past knowledge of the biosynthetic machinery and its regulation, or global screens that combine classical approaches of strain evolution through adaptation and selection with –omics approaches that can globally assess changes leading to desirable outputs.

Efforts to produce terpenoids in S. *cerevisiae* are characteristic of the variety of novel approaches used for strain improvement. Terpenoids and isoprenoids are an important class of secondary metabolites contributing more than 50,000 compounds to the rich chemical diversity of natural product structures [2]. Members of this group have attracted commercial interest as flavour and fragrance additives in the food and cosmetic industry. One such example is sclareol, an industrially important diterpene used by the fragrance industry [3, 4]. Many terpenoids also possess pharmaceutical properties and are currently used in clinical practice. Among them taxol, a diterpene from yew, which has successfully been established as a major antineoplastic agent, and artemisinin, a sesquiterpene lactone, which is an effective antimalarial agent [5–10]. Recently, attention has also focused on microbially produced terpenes as biodiesel [11–13].

Terpenoids are biosynthesized from two C_5_ precursors, isopentenyl diphosphate (IPP) and dimethylallyl diphosphate (DMAPP) [14]. In yeast and mammals, IPP originates from acetyl-CoA through the intermediate mevalonic acid (MVA). IPP then gives rise through the action of prenyltransferase enzymes to the higher order building blocks, geranyl pyrophosphate (GPP; C_10_), farnesyl pyrophosphate (FPP; C_15_) and geranylgeranyl pyrophosphate (GGPP; C_20_) [14]. In yeast, most of the pathway output in the form of FPP is utilized for the biosynthesis of sterols. Sterols are essential structural and regulatory components of eukaryotic cell membranes. Ergosterol, the main sterol in yeast, is responsible for structural membrane features such as fluidity and permeability, in a similar way as cholesterol is in human cells [15]. The pathway has been extensively studied for several years, since it functioned as a model for understanding human disease caused by high cholesterol levels [16] and is target of an important class of antifungal compounds [16]. Extensive knowledge on the biosynthesis and regulation of the pathway provided the first set of targeted interventions to increase the pool of the intermediates GPP, FPP, and GGPP, which are the substrates of terpene synthases.

The terpene hydrocarbon scaffolds are generated by the action of mono-, sesqui-, and diterpene synthases that catalyze multi-step reactions with diphosphorylated substrates of 10 (GPP), 15 (FPP) or 20 (GGPP) carbon atoms. The reactions catalyzed by terpene synthases are unparalleled relative to other classes of enzymes because they often consist of a series of stereochemically complex steps. These reactions initiate by the ionization of the diphosphate substrate to create an acyclic and reactive carbocationic intermediate. Subsequent regio- and stereo-specific formation of single or multiple rings, proton eliminations to form double bonds, water quenching of carbocations to create terpene alcohols, and stereospecific hydride, methyl, and methylene migrations, give rise to a vast complexity of structures [17, 18]. All this chemical complexity is catalyzed by enzymes whose three dimensional structure is highly conserved from fungi to plants, characterized by an active site lined mainly by inert amino acids [18]. Yeast does not normally produce terpenoids. However, expression of plant derived terpene synthases in yeast cells showed that it was possible for the enzymes to utilize the endogenous substrates (GPP, FPP, GGPP) and produce a range of terpenoid compounds [9, 19, 20]. The number of terpenoids produced is continuously growing as new genes from plant sources are cloned and characterized [21–26]. Following the formation of the olefin structure of terpenes, the metabolites can be further modified in nature by various cytochrome P450-dependent mono-oxygenases (P450), reductases, dehydrogenases or various classes of transferases, which expands immensely the variety of chemical structures synthesized [27]. Yeast, is the preferred host for P450 expression as it can express functionally active plant derived P450 enzymes [9, 28–30].

## Targeted interventions in the yeast MVA pathway to increase the terpene substrate pool

### HMGR

**Figure 1:**
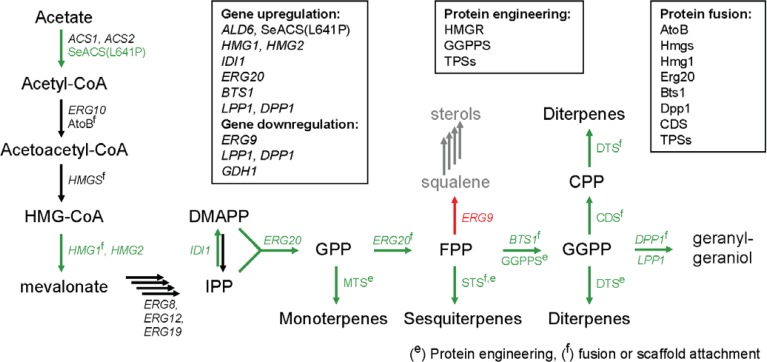
**Synopsis of terpene biosynthesis in yeast indicating the genes involved and the metabolic engineering interventions employed.** Upregulated yeast genes indicated indicated in green, downregulated yeast genes in red. Genes whose products have been fused or attached to a synthetic protein scaffold are denoted with supersripted (f). Enzymes with product yield or specificity improved or altered by protein engineering are indicated by superscripted (e). (CPP, copalyl diphosphate; CDS, copalyl diphosphate synthase; DTS, diterpene synthase; MTS, monoterpene synthase; SeACS(L641P), Salmonella enterica acetyl-CoA synthase mutant L641P; AtoB, acetoacetyl-CoA synthase/thiolase).

HMG-CoA reductase (3-hydroxy-3-methyl-glutaryl-CoA reductase; HMGR) is the key enzyme of the MVA pathway. The HMGR-catalyzed reaction produces mevalonic acid from HMG-CoA by reduction with NADPH (Figure 1). Statins, a class of top-selling drugs for lowering cholesterol, target HMGR [31]. Yeast possesses two enzymes of HMGR, Hmg1p and Hmg2p, which share a similar structure to the mammalian and plant counterparts composed of an N-terminal eight-helix spanning domain, a linker, and a C-terminal catalytic domain. Between the two enzymes Hmg1p is considered quite stable whereas Hmg2p undergoes mevalonate products-induced degradation [32]. A variety of early studies had pointed out that increased levels of pathway products led to degradation of Hmg proteins, lowering production levels [33, 34]. As a first step towards sterol-overproducing strains, Polakowski and co-workers used a deregulated version of Hmg1p [35]. The gene was truncated and the soluble tHmg1p catalytic domain was overexpressed, causing accumulation of a large amount of squalene [35]. Overexpression of t*HMG1* was applied to improve amorphadiene levels produced in yeast [9]. It has since become a basic tool for intervention to the MVA pathway, either by expressing t*HMG1* episomally or by integrating it into the chromosome [10, 25, 36, 37]. Alternatively, our lab used a stable variant of Hmg2p which harbors a point mutation substituting Lysine 6 by an Arginine (K6R), thus rendering the enzyme resistant to ubiquitination. Expression of *HMG2* (K6R) had equivalent effects in enhancing monoterpene and sesquiterpene production [21]. The extent by which proper subcellular localization of Hmg proteins on the perinuclear membrane of the engineered strains may affect their function is not yet clear. However, we have recently identified a set of gene perturbations which lead to increased stabilization of the endogenous Hmg proteins and to dramatic improvements in sesquiterpene production that could not be achieved by tHmg2p overproduction alone (Ignea, submitted).

### ERG9

The *ERG9* gene codes for squalene synthase, the enzyme that joins two FPP moieties to form squalene in the first committed step of the sterol biosynthetic pathway. Squalene formation is the major draining route of isoprenoid substrates, and as such its suppression is desirable. Since deletion of *ERG9* is lethal, complete elimination of squalene synthesizing activity is not feasible. Studying Hmg2p regulation, Gardner and Hampton [38], used a tunable *MET3* promoter (P_MET3_) to replace the endogenous *ERG9* promoter. Addition of methionine in the medium at concentrations >0.5 mM suppressed the transcription of *ERG9* [32]. The same approach was taken by Ro et al. [9], who used *ERG9* suppression, under conditions of t*HMG1* overexpression, to achieve an additional 2-fold improvement in the levels of amorphadiene production. Suppression of *ERG9* through the use of a P_MET3_ promoter was also used in an effort to increase production of the sesquiterpenes cubebol, valencene and patchoulol [33]. In this study, the introduction of the P_MET3_ promoter was less efficient than anticipated and a concomitant increase in farnesol formation, as by-product, was observed. This raised the issue of tightness of P_MET3_ regulation due to consumption of methionine in the medium [20]. A recent study addressed this issue by testing fusions of the P_MET3_ to the lacZ gene and monitored *β*-galactosidase activity of the strain growing under different regiments of methionine supplementation [24]. In the cultures that contained L-methionine, LacZ activity was initially very low. However, at about mid-exponential phase, it started to increase and rapidly reached levels measured in the non-repressed cultures [24]. To provide a tighter control of *ERG9* expression, the glucose regulated promoters P_HTX1_ and P_HTX_2 were tested, aiming to maintain moderate expression of *ERG9* during exponential growth and maximal repression during glucose limited conditions in a batch cultivation process. P_HTX1_ was shown to be efficient in downregulating *ERG9* expression under glucose limiting conditions. An alternative to using promoters with varying strengths and inducibility was based on posttranscriptional mechanisms controlling mRNA stability [39]. A set of hairpin sequence modules recognized by the RNAase III enzyme Rnt1p can be inserted at the 3'UTR of a gene to regulate transcript stability. With decreasing transcript levels, a similar decrease in protein levels was observed. This strategy was tested on *ERG9* and depending on the hairpin module used, achieved a level of suppression ranging between 40%-100% of control transcript levels. In an additional approach to suppress *ERG9* transcript levels, our lab tested the heterozygous deletion of the gene in a diploid strain, aiming to capitalize on previous observations revealing that in yeast loss of the one allele leads in the vast majority of cases to almost 50% reduction in protein levels [40]. Co-expression of *HMG2*(K6R) together with *erg9/ERG9* haploinsufficiency increased the production of monoterpenes and sesquiterpenes by >4-fold [21]. The approach does not require complex media and could be used in a continuous fermentation system. It can also be applied to numerous other genes, thus downregulating competing biochemical pathways or alleviating regulatory restrictions. It would be interesting to test P*HTX1* regulation in the remaining allele (P*HTX1*-*ERG9/erg9*) as the level of repression would be tighter.

### ERG20

The Erg20p enzyme catalyzes the condensation of IPP and DMAPP to form GPP and subsequently FPP, which is the major product. *ERG20* overexpression was used to increase amorphadiene production but its effect was limited to a 10% increase in total yield [9]. A small increase in sesquiterpene production was also observed when one allele of *ERG20* was controlled by the strong P_GAL1_ [21]. The ratio of GPP:FPP produced in vitro by a cell extract of wild type cells was 25:75. A mutation was identified in lysine 197 of Erg20p (K197E) which suppressed the second part of the catalysis resulting in reversed ratios 70:30 GPP:FPP [41]. This shift towards GPP formation was used to enhance monoterpene formation in yeast cells. Expression of geraniol synthase in an *ERG20* (K197G) background resulted in important improvement in geraniol productivity [42].

### ACS

Shiba *et al*. addressed the bottleneck in the supply of acetyl-CoA to the mevalonate pathway, and showed that overproduction of acetaldehyde dehydrogenase *ALD6* and introduction of a heterologous acetyl-CoA synthase variant from *Salmonella enterica* (L641P) together with t*HMG1* expression achieved substantial improvements in amorphadiene production [43].

### UPC2

Upc2p and Ecm22p are two highly homologous zinc cluster proteins regulating a number of ERG genes in the yeast ergosterol biosynthetic pathway. They positively regulate transcription by binding to sterol response elements in the promoters of the target genes. The upc2-1 mutant contains a single amino acid change (G888D) within the activation domain of the protein [44, 45]. Overexpression of *upc2-1* by itself appears to exert modest effects on amorphadiene production which become more pronounced in combination with t*HMG1* and P_MET3_-*ERG9* [9–11]}.

### IDI1

Encodes for IPP isomerase, catalyzing the conversion of IPP to its isomer DMAPP. *ERG20* adds one molecule of IPP to DMAPP to form GPP. In the case of monoterpene production, when a rich GPP pool is required, *IDI1* overexpression significantly enhanced monoterpene titers [21].

### BTS1

Encodes for geranylgeranyl diphosphate synthase. The enzyme uses FPP and IPP to synthesize the C_20_ GGPP substrate used for ubiquinone biosynthesis and geranylgeranylation of proteins for membrane attachment. GGPP is the substrate for diterpene and carotenoid biosynthesis. Overexpression of BTS1 has been combined with CrtYB and CrtI from *Xanthophyllomyces dendrorhous* to generate *β*-carotene and with CrtS to produce astaxanthin [46, 47]. Bts1p has been fused to Erg20p to improve product yields for geranylgeraniol [48, 49] and miltiradiene [25]. When the crtE gene, which encodes for the GGPP synthase of X. *dendrorhous*, was co-expressed with CrtYB and CrtI, significant improvement over BTS1 was observed [47]. This does not appear to be due to species specificity of GGPPS, since we observed a comparably high production upon co-expression of CrtYB and CrtI with the plant derived *Cistus creticus* GGPPS1 [50] (unpublished observations).

### LPP1, DPP1

Inhibition of squalene synthase (SQS) in mammalian species was shown to lead to conversion of FPP to farnesol. Farnesol accumulation was also seen in yeast strains treated with zaragosic acid, a natural inhibitor of SQS [51]. It has been postulated that dephosphorylation of FPP and GPP may be a mechanism to alleviate the potentially toxic effects of substrate accumulation. Lpp1p and Dpp1p, two enzymes initially identified as phosphatidic acid hydrolases were shown to also dephosphorylate isoprenoid phosphates [52]. Deletion of *DPP1* was reported to result in a modest increase of the sesquiterpene *α*-santalene and a 24% drop in farnesol accumulation [24]. However, other studies aiming at high sesquiterpene production did not observe significant improvements [53, 54]. Still, when *LPP1* and *DPP1* were overexpressed fused to *BTS1*, they exerted a strong positive effect on geranylgeraniol production, with *DPP1* exerting the strongest effect, yielding 2.9-fold higher levels of GGOH than simple co-expression of the genes [49].

## Protein engineering to improve product yield and to expand the chemical diversity

The primary goals of protein engineering studies to date have been to increase product yield and to interfere with the cyclization chemistry of the terpene synthases so as to either produce enzymes with higher specificity or to derive new products out of a specific enzyme.

*Improving product yield* – Based on the previous observation that the two main bottlenecks in terpene biosynthesis in *E. coli* are caused by the poor activities of the HMGR and the terpene synthase [55], Yoshikuni and coworkers developed a methodology based on adaptive evolution to improve the catalytic efficiency of these two enzymes [56]. By comparing the sequences of a large number of central metabolic enzymes, they noticed that Gly and Pro were significantly less frequently mutated than other amino acids in *E. coli* enzymes, suggesting that these residues may be more essential. They applied this finding in the truncated form of HMGR and in an inefficient humulene synthase. By switching non-conforming Gly and Pro residues to match the consensus sequence, and by saturation mutagenesis of the glycines and prolines that did not align, an engineered version of tHMGR (tHMGR-G9), which contains nine mutated residues, exhibited a 2.5- to 3-fold increase in the production of mevalonate compared to the wild-type tHMGR. Similarly, HUM-G6 (an engineered version of humulene synthase bearing six such substitutions) improved sesquiterpene production by 80 fold. Integration of the tHMGR-G9 and HUM-G6 mutants into the same host resulted into a three- to four-fold improvement in growth, leading to an overall improvement in sesquiterpene production by nearly 1000 fold [56].

Using a colorimetric assay based on the production of lycopene in *E. coli* as a high-throughput screening method, Leonard and co-workers applied a saturation mutagenesis approach to isolate improved Taxus canadensis GGPPS variants [57]. The double mutant S239C/G295D improved levopimaradiene production by 1.7-fold. To further improve levopimaradiene production, fifteen residues constituting the binding pocket of levopimaradiene synthase (LPS) were selected based on homology modeling. Perturbation of these residues using phylogeny-based mutation, saturating mutagenesis, and combination of the beneficial mutations improved enzyme productivity by up to 10-fold (some combinations also showed an improvement in product specificity, albeit with lower than maximal productivity) [57].

*Improving product specificity* – To investigate whether a promiscuous terpene cyclase can be engineered to have improved product selectivity, Yoshikuni and coworkers employed γ-humulene synthase from *Abies grandis*, an enzyme with an extremely promiscuous product profile, synthesizing more than 50 different sesquiterpene products. Using homology modeling, they identified 19 residues which were subsequently altered by saturation mutagenesis. By systematically combining different beneficial mutations, a collection of more specific terpene synthases was constructed, including an E-*β*-farnesene synthase, a siberene synthase, a *β*-bisabolene synthase, a longifolene synthase and an *α*-longipinene synthase [58].

*Expanding product diversity* - Until recently, there has been insufficient evidence to support the conclusion that a modified terpene synthase scaffold can produce entirely novel compounds, rather than altered levels of compounds already made by the parent scaffold. Although the large scale mutagenesis of *A. grandis* γ-humulene synthase mentioned above yielded mutant synthases with narrower product specificity, it failed to produce structures not synthesized by the wild-type enzyme [58]. However, this approach used a mathematical model to predict changes that would improve specificity for certain compounds and this may have influenced this screen against the identification of new entities. The first evidence for the ability of a terpene synthase scaffold to acquire a new specificity came through a concentric mutagenesis approach aiming to interconvert the activities of tobacco 5-epi-aristolochene synthase (TEAS) and *Hyoscyamus muticus* premnaspirodiene synthase (HPS). In this study, a combination of second tier residue alterations enabled the production of epi-aristolochene by HPS, an activity not present in the wild-type enzyme [59]. Our work on the engineering of Salvia terpene synthases extended this finding, by demonstrating that a monoterpene synthase can be modified to produce sesquiterpenes by a single amino acid substitution [60]. In the same study, the *Salvia pomifera* sabinene synthase 1 (Sp-SabS1) was modified to produce 1,8-cineole and a-terpineol, two products not made by the wild-type enzyme. Subsequent studies extended these observations to diterpene synthases, where single residue substitutions converted an isokaurene synthase into a specific pimaradiene synthase [61], or a levopimaradiene/abietadiene synthase into an isopimaradiene synthase [62]. The limited sequence-space explored in these experiments makes it possible that significant novel activity can be produced using more thorough mutagenesis approaches.

## Metabolic channeling by fusion enzymes

The generation of fused enzymes has been proposed as a means to minimize losses of intermediate products through diffusion, degradation, or utilization by rival enzymes. This could be especially important when exogenous enzymes, such as terpene synthases (TPSs), are used. Fusions of Erg20p to TPSs were tested for the production of patchoulol and exhibited higher yields than the separate enzymes [53]. Not all fusions appear to result in a net gain, since some enzymes are also sensitive to their placement in the fusion. We noted that a C-terminal fusion of EYFP to a sesquiterpene synthase (STS) caused a significant reduction in enzymatic activity. Similarly, no improvement was seen when farnesyl diphosphate synthase (FPPS) was fused at the C-terminus of patchoulol synthase (PTS), in contrast to the inverse fusion (FPPS-PTS) [53]. Expanding this system, a dual fusion strategy was employed by Zhou and colleagues for the production of the diterpene miltradiene. The Bts1 protein was fused to the N-terminus of Erg20p to drive efficient GGPP production, while copalyl diphosphate (CPP) synthase was appended to the C-terminus of miltiradiene synthase to provide the latter with ample adequate CPP substrate [25]. A parallel approach fused FPPS and STS genes to the COX4 mitochondrial targeting sequence, aiming to target the pathway to the organelles where FPP pools are naturally present. When combined with cytosolic tHMG1 overexpression, an extra boost in sesquiterpene production was observed [63].

An alternative approach to fusion proteins was applied to the mevalonate pathway by attachment of protein interaction domains and their respective peptide ligands. Expression of yeast HMGR and HMG-CoA synthase (HMGS) attached to the SH3 domain and the SH3 ligand respectively in *E. coli* improved mevalonate yields [64]. The protein interaction domains were also built into a synthetic scaffold module (GBD-SH3-PDZ domains) and AtoB (Acetoacetyl-CoA thiolase, native to *E. coli*), HMGS and HMGR were fused to the corresponding peptide ligands. This co-recruitment of proteins increased production of mevalonate dramatically (77-fold) [64]. As the number of terpene decorating enzymes introduced in yeast increases, the scaffold approach could provide the desirable biosynthetic pathway efficiency.

## Coping with stress

Increased production of terpenes, especially oxidized compounds is expected to impose stress on the cellular machinery. Examination of cells producing artemisinic acid for induction of pleiotropic drug resistance revealed high induction of the ABC transporters *PDR5, PDR15, YOR1*, and *SNQ2* [65]. Identification of plant-pathway components which participate in biosynthesis and transport of terpenoids could be an additional means to relieve the stress imposed on the yeast cell by high production. We previously identified a plant HSP90 by two-hybrid assays, which interacted with the monoterpene cineole synthase (SfCinS1). Co-expression of the HSP90 and SfCinS1 in yeast cells contributed to a modest, albeit consistent, improvement in cineole production [21]. Presumably additional plant genes can be tested for their contribution to terpene biosynthesis in yeast cells.

## Global approaches to strain improvement

The development of –omics technologies and advanced modeling tools has enabled global approaches which could illuminate the genetic basis of phenotypic diversity. Such an approach was applied to assess the differences in ergosterol production between widely used lab strains. One such strain is CEN.PK113-7D which is becoming a strain of choice for terpenoid production. The strain was fully sequenced, annotated and compared to the 288c reference genome to identify single nucleotide polymorphisms (SNP) which could be informative on the nature of changes taken place in the ergosterol pathway. Focusing only on metabolic genes, 85 out of 219 SNPs were encoding for amino acid changes (non-synonymous or non-silent) [66]. A number of SNPs were identified in the genes ERG8 (phosphomevalonate kinase), *ERG9* and *HFA1* (mitochondrial acetyl co-enzyme A carboxylase catalyzing the production of malonyl-CoA in fatty acid biosynthesis). Co-expressed with *β*-amyrin synthase with combinations of these variants led to a 5-fold improvement in amyrin production [67].

Another computational approach based on a minimization of metabolic adjustments algorithm (MOMA), identified *GDH1* as a possible target which could shift the metabolic flux towards the ergosterol pathway. The gene encodes a glutamate dehydrogenase involved in ammonium metabolism in yeast and requires NADPH for its function. The conversion of HMG-CoA to mevalonate is an NADPH requiring step, thus deletion of *GDH1* was postulated to be beneficial for carbon flux through the mevalonate pathway by increasing the pool of available NADPH for HMGR. In yeast there are two other glutamate dehydrogenase enzymes encoded by *GDH2* and *GDH3. GDH3* appears to have arisen from genome duplication of *GDH1*, while *GDH2*, unlike the other two, is an NADH-dependent enzyme. Deletion of *GDH1* in cells expressing cubebol synthase led to approximately 85% increase in the final titer [68]. However, despite the presence of the other two GDH enzymes, *gdh1* causes severe growth impediments [68]. An approach to suppress but not eliminate protein levels could be effective without the side effects. Recently, we tested heterozygous deletions of *GDH1* alone or in combinations with other haploinsufficiencies and observed a modest but consistent increase in sesquiterpene production (Ignea, submitted). Being complementary to approaches focused on the biosynthetic components, as the production yield increases, suppression of *GDH1* may become more important.

The dramatic reductions in sequencing costs and the rapid development of new computational tools will contribute to classical strain improvement approaches using evolution and adaptation by providing feedback on the genetic changes that take place and cannot be assessed by metabolic flux models or rational design approaches.
